# Gene Expression Profile Analysis is Directly Affected by the Selected Reference Gene: The Case of Leaf-Cutting *Atta Sexdens*

**DOI:** 10.3390/insects9010018

**Published:** 2018-02-08

**Authors:** Kalynka G. do Livramento, Natália C. Freitas, Wesley P. F. Máximo, Ronald Zanetti, Luciano V. Paiva

**Affiliations:** 1 Central Laboratory of Molecular Biology, Federal University of Lavras (UFLA), Lavras, MG 37200-000, Brazil; nataliabiotec@gmail.com (N.C.F.); wesleypfmax@posgrad.ufla.br (W.P.F.M.); 2 Entomology Department, Federal University of Lavras (UFLA), Lavras, MG 37200-000, Brazil; zanetti@den.ufla.br; 3 Chemistry Department, Federal University of Lavras (UFLA), Lavras, MG 37200-000, Brazil; luciano@dqi.ufla.br

**Keywords:** ant, endogenous controls, normalization, quantitative RT-PCR, RefFinder, *SNF7*, validation

## Abstract

Although several ant species are important targets for the development of molecular control strategies, only a few studies focus on identifying and validating reference genes for quantitative reverse transcription polymerase chain reaction (RT-qPCR) data normalization. We provide here an extensive study to identify and validate suitable reference genes for gene expression analysis in the ant *Atta sexdens*, a threatening agricultural pest in South America. The optimal number of reference genes varies according to each sample and the result generated by RefFinder differed about which is the most suitable reference gene. Results suggest that the *RPS16*, *NADH* and *SDHB* genes were the best reference genes in the sample pool according to stability values. The *SNF7* gene expression pattern was stable in all evaluated sample set. In contrast, when using less stable reference genes for normalization a large variability in *SNF7* gene expression was recorded. There is no universal reference gene suitable for all conditions under analysis, since these genes can also participate in different cellular functions, thus requiring a systematic validation of possible reference genes for each specific condition. The choice of reference genes on *SNF7* gene normalization confirmed that unstable reference genes might drastically change the expression profile analysis of target candidate genes.

## 1. Introduction

*Atta sexdens* leaf-cutting ant is considered the main eucalyptus plantations pest and for therefore, the need to find more viable pest control methods has proportionally increased for forest plantation expansion [1]. Such insect can cause total plant defoliation in *Eucalyptus* species, affecting both diameter and height of trees, hence leading to a production decrease and consequent profit reduction [2]. Currently, the major strategy used to control leaf-cutting ant has been through chemical insecticides-based methods which are very toxic and have had its applications restricted by Kyoto protocol. The RNA-mediated interference (RNAi) mechanism via double-stranded RNA (dsRNA) has been a potential alternative not only to combat pest insects [3,4] but also to study gene expression in ants [5]. Regardless of the choice, it is necessary to evaluate the expression levels of target genes by quantifying transcribed RNAs at a given time, specific condition through quantitative reverse transcription polymerase chain reaction (RT-qPCR).

RT-qPCR technique is both efficient and reproducible for gene expression quantification during real time. However, many factors may influence data normalization in RT-qPCR, such as quality and integrity of RNA samples as well as efficiency in complementary DNA (cDNA) synthesis [6]. In order to minimize these influences on experiments and at the same time ensure their reproducibility, data normalization from gene expression analysis of reference genes is commonly employed since both reference and target genes can be quantified in the same samples [7,8].

The reference gene choice has to be thoroughly made, hence, picking endogenous genes that express quantitatively themselves in all cells regardless of conditions and stimuli [9]. RefFinder (East Carolina University, Greenville, NC, USA) is currently the most promising evaluation tool for reference genes selection as provides the ranking of genes based on results from four distinct softwares geNorm [7], NormFinder [10], BestKeeper [11] and Delta-Ct [12] and also calculates the geometric mean of their values for a final general ranking [13], generating more consistent and appropriate results for RT-qPCR analyses.

The statistics from geNorm and BestKeeper depends on that expression ratio of two ideal reference genes be constant among all samples regardless of experimental conditions. On the other hand, NormFinder considers that considers that expression stability average variations of multiple genes are lower than those observed in single genes expression and that reference genes involved with different groups may contribute to decrease such variations [14]. Gene classification using the Delta-Ct method is based on partial comparisons through crude values, taking into account that the mean standard deviation (*SD*) of each set of genes is inversely proportional to gene stability [12].

The reference gene stability by the geNorm software is calculated from an *M* value, defined as the average expression variation of a given gene over all others tested. Genes with the lowest *M* values have the most stable expression [7] and *M* values equal to 1.5 are generally considered cut-off values [15,16,17]. The NormFinder software is based on analysis of variance and allows estimating the variation value of intra and inter-sample gene expression, as well as the calculation of expression stability (*SV*) values for candidate reference genes. Genes with lower *SV* are considered more stable and show the lowest variation combining intra and inter-sample expression [10]. BestKeeper software individually evaluates the gene expression stability for all genes based on three variables: standard deviation (*SD*), correlation coefficient (*r*) and percentage of covariance (*PC*) [11]. The Delta-Ct analyzes the variability index among *Cq* values of samples and, at the end, the lowest value is considered the most stable [12]. The NormFinder software is based on analysis of variance and allows estimating the variation value of intra and inter-sample gene expression, as well as the calculation of expression stability (*SV*) values for candidate reference genes. Genes with lower *SV* are considered more stable and show the lowest variation combining intra and inter-sample expression [10]. BestKeeper software evaluates the gene expression stability for all genes individually based on three variables: standard deviation (*SD*), correlation coefficient (*r*) and percentage of covariance (*PC*) [11]. The Delta-Ct analyzes the variability index among *Cq* values of samples and, at the end, the lowest value is considered the most stable [12].

This approach has been successfully applied on reference genes determination in a wide range of organisms including plants, insects and animals, such as *Coffea arabica* [18], *Solenopsis invicta* [19] and *Ovis aries* [20], respectively.

The aim of this study was to select a set of reference genes with stable expression in different *A. sexdens* samples based on the RefFinder results. The stability of candidate genes as ribosomal protein L18 (*RPL18*), ribosomal protein L32 (*RPL32*), ribosomal protein S16 (*RPS16*), glyceraldehyde-3-phosphate dehydrogenase (*GAPDH*), NADH dehydrogenase (*NADH*) and succinate dehydrogenase B (*SDHB*) was evaluated in different tissues (head and midgut of forager and larval midgut), development stages (foragers, larva and pupa) and castes (foragers, soldier and queen). As a result, different sets of reference genes were recommended according to each sample group, as well as optimal gene numbers. Finally, we validated the selection of our reference genes by evaluating the *SNF7* gene expression profile, involved in identifying and selecting proteins that will undergo lysosomal degradation [21].

## 2. Materials and Methods

### 2.1. Biological Samples

All samples came from three colonies of *A. sexdens* ants kept in the Laboratory of Integrated Pest Management, Department of Entomology, Federal University of Lavras, MG, Brazil. The ants were maintained at 23 ± 2 °C under relative humidity of 60 ± 10%. Ants were fed on *Acalypha hispida*, *Morus nigra* or *Hibiscus sabdariffa* leaves, once a day. 

Each biological triplicate consisted of 25 individuals (forager, soldier and pupa) from one colony. The biological triplicates from “larva” (75 larva) and “head” samples (75 heads) were collected by using surgical scissors and immediately placed in liquid nitrogen for maceration. Midgut of foragers and larva (200 units per replicate) were removed under a magnifying glass Stemi 2000 (Zeiss, Thornwood, NY, USA) model and directly macerated in TRIzol^®^ Reagent (Invitrogen, Carlsbad, CA, USA). The queens were randomly collected upon flock and after wing drop. Each biological sample consisted of 25 individuals stored at −80 °C in a 50 mL falcon tube for further maceration.

### 2.2. Total RNA Extraction and cDNA Synthesis

Total RNA was extracted using TRIzol^®^ Reagent (Invitrogen, Carlsbad, CA, US), dissolved in RNase-free water and stored at −80 °C. The RNA integrity was determined by denaturing agarose gel (1.2%) electrophoresis in 1× TAE buffer (0.04 M Tris-acetate, 0.001 M EDTA (pH 8.0)) and stained by ethidium bromide (EtBr). The intense ribosomal RNA bands with absence of smears after electrophoresis confirmed the RNA integrity. The RNA concentration of each sample was measured in triplicate using a Nanodrop 1000 spectrophotometer (ThermoFischer Scientific, Waltham, MA, USA). The RNA purity was measured by the 260/280 nm ratio, with expected values between 1.8 and 2.0. 

Thereafter, the RNA samples were treated with DNase (TURBO^TM^ DNase-Ambion, Waltham, MA, USA), according to the manufacturer’s recommendations. cDNAs were synthesized from 800 ng of total RNA in triplicate by using the High-Capacity cDNA Reverse Transcription kits (Applied Biosystems-Life Technologies, Waltham, MA, USA) following the manufacturer’s recommendations. The cDNA quality was confirmed by the *RPL18* gene (108 bp) amplification followed by 1.0% agarose gel electrophoresis using 10 μL of its PCR product. The synthesized cDNAs were stored at −80 °C for further RT-qPCR. 

### 2.3. Selection of Candidate Reference Genes and Primer Design

A set of six candidate genes were selected comprising several conventionally used reference genes in insects and other species based on previous reports [22,23,24] such as: ribosomal protein L18 (*RPL18*), ribosomal protein L32 (*RPL32*), ribosomal protein S16 (*RPS16*), glyceraldehyde-3-phosphate dehydrogenase (*GAPDH*), NADH dehydrogenase (*NADH*) and succinate dehydrogenase B (*SDHB*).

Orthologous sequences search for candidate reference genes was performed through the Basic local alignment search tool (BLAST), using the available sequences of the genes under study on GenBank (http://www.ncbi.nlm.nih.gov/) and in the ant *Atta cephalotes* genome (http://www.antgenomes.org) (Table 1).

The sequences were submitted to Primer Express software version 3.0 (Applied Bio-systems) for primer design. The length of the primers was between 20 and 22 bp with GC content ranging from 45% to 60% and a melting temperature (Tm value) in a range from 57 to 63 °C.

The primer pair specificity was verified through the dissociation (melting) curve analysis. Both PCR amplification efficiency (*E*) and regression coefficient (*R*^2^) were determined during the validation of primers according to the standard curve method using a set of all cDNA samples with 5× serial dilution. The specifications of the selected reference genes and primer pairs are shown on Table 1.

### 2.4. RT-qPCR Amplification

RT-qPCR analyses were performed using the Applied Biosystems 7500 Real-Time PCR system with a reaction mix containing 5.0 μL of SYBR^®^ Green PCR Master Mix (Applied Biosystems, Foster City, CA, USA), 1.0 μL of cDNA, optimized concentrations of primers (see Table 1) and RNase-free water to a total volume of 10.0 μL. Amplification conditions were: initial denaturation at 95 °C for 10 min and 40 cycles of denaturation at 95 °C for 15 s with both annealing and extension steps at 60 °C for 1 min. In order to confirm the primer specificity, melting curves were recorded after the 40 amplification cycles had been completed by increasing the temperature from 60 to 95 °C. All RT-qPCR assays were carried out in technical and biological triplicate.

### 2.5. Expression Stability Analysis of Candidate Reference Genes

The expression levels of candidate reference genes were determined based on the quantification cycle (*Cq*), also known as the threshold cycle (*Ct*), which is defined as the cycle at which the amplification fluorescence exceeds the one coming from the background [25]. The *Cq* values were determined by using 7500 software version 2.0.5 (Applied Biosystems, Foster City, CA, USA) and corrected according to the efficiency of each primer pair [18]. Box plot diagrams were made using Microsoft Excel 2013 to illustrate levels and variations in expression of each tested reference gene. 

The RefFinder tool (http://150.216.56.64/referencegene.php) was employed to assess the gene expression stability in four sample sets: (i) caste (queen, forager and soldier), (ii) development stages (larva, pupa and forager) (iii) tissues (forager head, larval midgut and forager midgut) and (iv) a pool of biological samples representing all sample types from (i) to (iii). 

### 2.6. Determination of the Minimum Number of Reference Genes

Based on the rank order obtained after RefFinder analyses, pairwise variations (*V*-values) were calculated for each dataset to establish the minimum number of reference genes needed for accurate data normalization. In summary, *Vn*/*n* + 1 is calculated between each set of two sequential normalization factors (*NF*) (starting with the relative expression values of the two most stable genes, as ordered by RefFinder) for all samples in each dataset. 

An array consisting of the log2-transformed *NF* ratios of every sequential combination of two *NF* in each sample is calculated. Finally, the standard deviation (*SD*) of the array data for each *NF* combination is calculated (*Vn*/*n* + 1) and plotted to display changes in expression stability of *NF* in comparison to the employed number of genes [6]. 

### 2.7. Validation of Reference Genes by SNF7 Expression Analysis

To verify how the expression data normalization for a gene of interest is affected by employing different reference genes, using both the most-stable reference genes and the most-unstable ones, the same eight cDNA samples used for the stability analyses of reference genes were also analyzed by RT-qPCR and then calculated by applying Pfaffl formula [26] for the *SNF7* gene expression. 

## 3. Results

### 3.1. Primer Specificity and Efficiency

Primer specificity during PCR amplifications was confirmed by the presence of a single peak on the melting curve.

The PCR efficiency (*E*) and the regression coefficient (*R*^2^) were calculated using the slope of the standard curve established for each primer pair. *E*-values ranged from 90% to 102% and *R*^2^ showed values equal to or greater than 0.973 (Table 1), indicating that template cDNA was successfully duplicated at the end of each cycle.

### 3.2. Expression Profile of Candidate Reference Genes

**Figure 1:**
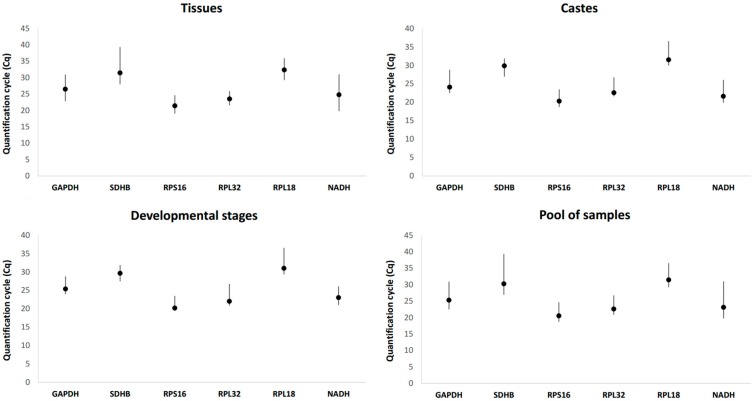
Expression of candidate reference genes as determined by the quantification cycle (*Cq*) values determined in four sample sets. *Bars* indicate maximum and minimum *Cq* values while *circles* represent mean values.

The expression levels and variations of the tested genes were analyzed for sample sets: caste (Queen, forager and soldier), development stages (larva, pupa and forager), tissues (forager head, larval midgut and forager midgut) and a pool of biological samples representing all sample types from caste, development stages and tissues (Figure 1). The six potential reference genes showed expression with a wide range of *Cq* values (18.7 to 39.4).

In the sample pool analysis, *RPL18* showed the lowest expression level with an average *Cq* value equivalent to 31.5 cycles, unlike *RPS16* which showed the highest expression level with an average value of 20.6 cycles. All candidate reference genes showed average *Cq* values ranging from 20 to 31 cycles. The highest expression variations among all tested samples were verified in the *SDHB* and *NADH* genes (Δ*Cq* = 12.4 and 11.3, respectively) and the lowest in the *RPS16* and *RPL32* genes (Δ*Cq* = 5.9 and 5.8, respectively). Δ*Cq* represents the variation between the maximum and minimum *Cq* for each gene.

By analyzing the different collected tissues (forager head, larval midgut and forager midgut), the expression levels ranged from 19.8 to 39.4 cycles. The most expressed gene was *RPS16* with average *Cq* value equivalent to 21.4 cycles. *SDHB* was the lowest expressed gene with an average *Cq* value of 31.5 cycles. In general, the highest expression of each candidate gene was observed in the “forager head” samples, except for the *RPL32* gene. This gene had the lowest variation in the expressions among all samples (Δ*Cq* = 4.4). The opposite was observed in *SDHB* gene expression (Δ*Cq* = 11.4), which ranged from 28.0 to 39.4 cycles, being less expressed in larval midgut tissues. 

When the castes were analyzed, it was possible to observe that candidate genes showed the highest expression in soldier samples, except for the *RPL18* gene, which was more expressed in the queens. In this case, the variation in candidate gene expressions ranged from 18.7 to 36.6 cycles. The lowest expression (*Cq* of 31.5 cycles) was observed for the *RPL18* gene in forager ants, while the highest expression (*Cq* of 20.3 cycles) was in the *RPS16* gene in soldiers. In the forager ants, it was possible to verify the highest expression variations among all genes when castes were analyzed and the development stages when the samples were analyzed.

Forager, larva and pupa were the samples used to analyze the candidate reference gene expression during ant development stages. *RPS16* and *NADH* genes showed the lowest mean values of *Cq*, 21.1 and 23.4 cycles, respectively, in forager. In larva, the *RPS16* gene showed the highest expression, with average *Cq* of 19.7 cycles. The other three genes (*RPL18*, *GAPDH* and *SDHB*) were less expressed at the pupal stage. *RPL18* candidate gene showed the highest variation (Δ*Cq* = 7.2) among the samples from development stages, with expression profile between 29.3 and 36.6 cycles.

### 3.3. Expression Stabilities of Candidate Reference Genes

The geNorm, NormFinder, Bestkeeper and Delta-Ct software packages were used through the RefFinder tool. Besides generating the analyses of each program, RefFinder serves as means of comparison between candidate genes general classification. The RefFinder analysis is essential when algorithms generate frequently contrasting results.

The geNorm, NormFinder, Bestkeeper and Delta-Ct software packages were used through the RefFinder tool. Besides generating the analyses of each program, RefFinder serves as mean of comparison between candidate genes general classification. The RefFinder analysis is essential when algorithms generate frequently contrasting results.

In expression stability analysis of candidate reference genes on the sample pool (Table 2), the *M* values from geNorm software were lower than the stability cut-off value (1.5).

The most stable genes were *GAPDH* and *NADH* (*M* = 0.132) and the less stable genes were *RPL32* (*M* = 0.248) and *RPL18* (*M* = 0.234). NormFinder identified *RPS16* (*SV* = 0.056) as the most stable reference gene and *RPL32* (*SV* = 0.228) as the least stable. BestKeeper indicated the *SDHB* gene as the most stable candidate (*SD* = 0.168) and *RPL32* (*SD* = 0.247) as the least stable. The Delta-Ct method determined *RPS16* and *NADH* as the most stable genes, with stability values equal to 0.204 and 0.246, respectively. The *RPL32* gene displayed the lowest stability (0.275). The final ranking suggested that the most stable reference gene was *RPS16* (1.565) followed by *NADH* (2.060), while *RPL32* was the least stable gene (6.000).

For analysis of different tissues, the geNorm and BestKeeper software indicated the *RPL18* gene as the most stable, with stability values of 0.079 and 0.204, respectively. However, NormFinder and Delta-Ct indicated the *RPS16* gene as the most stable, with stability values equal to 0.073 and 0.243, respectively. *SDHB* gene was considered the least stable in all methods of analysis. The final ranking suggested that the most stable reference gene was *RPL18* (1.414), followed by *RPS16* (1.968) and the least stable genes were *SDHB* (5.233) and RPL32 (5.045) (Table 3).

Among the castes, the *RPS16* gene was considered the most stable in the Normfinder, BestKeeper and Delta-Ct software, with stability values of 0.106, 0.182 and 0.208, respectively. *GAPDH* and *NADH* were the most stable genes through geNorm analysis, with *M* = 0.122 and the least stable gene being *RPL18* (*M* = 0.237), also considered the least stable gene in the analyses of all other three software packages. The final ranking showed *RPS16* (1.316) as the most stable reference gene, followed by *NADH* (1.861), while the least stables were *RPL32* (4.229) and *RPL18* (6.000) (Table 4).

Analyses from NormFinder, BestKeeper and Delta-CT suggested that the *RPS16* and *NADH* genes were the most and least stable, respectively, after evaluating samples from development stages. In the same sample set, the analysis performed by geNorm showed that the most stable genes were *RPL32* and *RPL18* (*M* = 0.163) and, similar to outcomes from other software, the *NADH* gene was the least stable (0.308). The final ranking suggested that the most stable reference gene was *RPS16* (1.316) followed by *RPL32* (2.115), while the least stable genes were *SDHB* (4.162) and *NADH* (6.000) (Table 5).

### 3.4. Determination of the Minimum Number of Reference Genes

In order to generate more accurate and reliable gene expression results, putting stable reference genes together is essential when multiple reference genes are used [24]. Normalization with an inappropriate number of reference genes can lead to significant analysis errors [7]. The optimal number of reference genes to be used for a more accurate normalization is determined by calculating *V*-values as a pairwise variation (*Vn*/*Vn* + 1) between two consecutively ranked normalization factors (*NF*) after the stepwise addition of the subsequent more stable reference gene (*NFn* and *NFn* + 1) [7], which is included in the geNorm package. The n indicates the number of the most stable reference genes.

**Figure 2:**
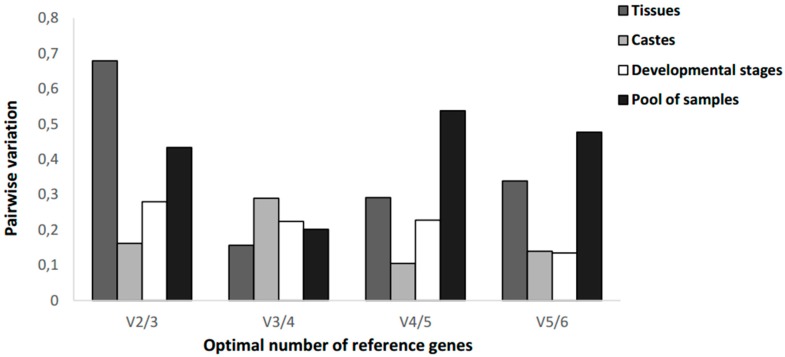
Pairwise (*V*) variation calculated by geNorm to determine the optimal number of reference genes.

In our study results showed that pairwise variation value for *V*3/4 was the lowest (0.20), indicating that the combination among three most stable reference genes would be sufficient for the gene expression normalization within total sample pool (Figure 2).

For gene expression analysis in different tissues (forager head, larval midgut and forager midgut), the pairwise variation value for *V*3/4 was the lowest (0.156), indicating that the combination among three most stable reference genes would also be sufficient for normalization in different tissue samples. 

The ideal gene expression normalization in caste samples (forager, soldier and queen) was obtained using the four most stable reference genes, on behalf of the Pairwise variation values for *V*4/5 to be equal to 0.105. 

Finally, for gene expression analysis at the development stages (larva, pupa and forager), the pairwise variation values for *V*5/6 were the lowest (0.134), indicating that the combination among five most stable reference genes would be sufficient for the gene expression normalization. 

The threshold variation value in pairs of 0.15 as demonstrated in the Vandesompele et al. (2002) [7] studies should not be rigorous and trend to change *V*-values is recognized as being equally effectual [27]. Moreover, normalization patterns indicate the use from two to five stable and validated reference genes as the most appropriate approach to normalize RT-qPCR data [28]. We have observed that the variability decrease obtained by adding a high number of reference genes does not overcome some disadvantages related to time consumption and additional costs after such inclusions.

### 3.5. Validation of the Selected Reference Genes

**Figure 3:**
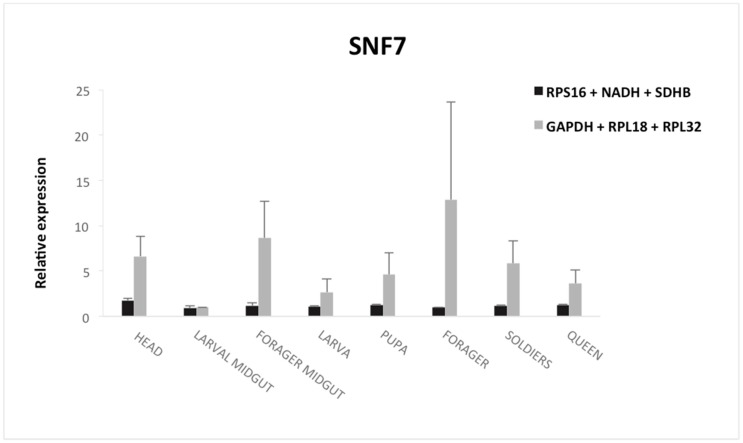
Differential gene expression of *SNF-7* using the selected reference gene. Relative gene expression quantification was performed using two different normalization strategies: the combination of the three top ranked genes and combination three most unstable genes. The *columns* represent the gene expression in different samples (head, larval midgut, forager midgut, larva, pupa, forager, soldiers and queen) of *Atta sexdens. Error bars* indicate standard deviation (SD).

To evaluate the reference gene selection impact on gene expression measurements, we analyzed *SNF7* gene expression using two normalization strategies: the combination of the three most stable genes (*RPS16*, *NADH* and *SDHB*) or the three most unstable (*RPL32*, *RPL18* and *GAPDH*) in the sample pool (Figure 3).

Relative abundance of transcripts for the target gene was dependent on reference genes used for normalization. The *SNF7* expression levels were over-estimated when using non-suitable reference genes for normalization. The *SNF7* expression pattern shows stable expression with a relative expression ratio around 1 in all samples. In contrast, when less stable genes were used for normalization, it was possible to verify a large variability in *SNF7* gene expression. Furthermore, identified normalization controls resulted in significantly lower standard deviations, ensuring greater reproducibility of results. Thus, indicating that questionable results could be produced if unstable reference genes had been used. These results show the importance of validating reference genes prior to experimental applications.

## 4. Discussion

Among the RT-qPCR applications, stand out the gene expression profile analysis, monitoring of primer efficiency and verification of gene knockdown through RNA interference-mediated functional effects, among others [29,30].

Regardless of the application, all experiments with RT-qPCR analyses should be standardized with more than one reference gene and these should be validated regarding its stability to avoid erroneous differences in expression [13]. In a recent research, RT-qPCR normalization procedures were analyzed in more than 1700 published papers and it was concluded that most articles had inadequate standardization procedures [31]. 

We have observed in some studies about gene expression in ants the use of only a single reference gene without any mention of its validation. Expression analyzes of genes involved in immunological responses, such as *PGRP-LB* and *PGRP-SC2* in the ant *Camponotus floridanus* were normalized with only one reference gene, the *RPL32* [32]. Similarly, the reference gene *β-actin* was the only one used for normalization of *Calreticulin* gene expression [33] and *Muscarinic Cholinergic receptor* [34] in studies with *Polyrhachis vicina.*

Although the need for systematic selection and validation of reference genes in RT-qPCR studies is widely requested, little information is available about the stability of reference gene expression in *A. sexdens*, even with genomes of some ant species already available on databases [35,36,37]. The increasing number of validation studies in transcriptome analyzes [38,39] and the works associated with gene silencing based on RNAi [5,40] promise the control monitoring of important agricultural pests affecting cultivated plant performance 

Our results suggest that the *RPS16*, *NADH* and *SDHB* genes were the best reference genes in the sample pool (head, larval and forager midgut, larva, pupa foragers and queen) according to stability values acquired by RefFinder, while the *RPL32*, *RPL18* and *GAPDH* genes were the least stable under the same analyses. Few differences were observed after analyzing the results of four software packages, except for the development stage samples (Table 2, Table 3, Table 4 and Table 5).

When we compared our results with the reference genes from *S. invicta* ant [19], it was observed that two analyzed genes are homologous to *A. sexdens* genes (*GAPDH* and *RPL18*). In both studies, *GAPDH* is among the least stable genes. In our work, *RPL18* was considered unstable to be used in normalization experiments of sample pool and castes in *A. sexdens*. In contrast, the expression of this gene was the second most stable in tissue samples and the third most stable in developmental stage. These results do not differ from those found in the reference gene evaluation in *A. sexdens rubropilosa*, on which *RPL18* was the most stable gene in tissues under development stage. [41].

In *Camponotus floridanus*, the homologues *RPL32*, *RPL18, EF1a* (elongation factor 1-alpha) and *GAPDH* were evaluated. For the *RPL32* gene, higher expression stability was observed in the analyses with pupa [42], in agreement with the evaluation performed on development stages in our study, in which the *RPL32* gene appears ranked in second place (Table 5). Using BestKeeper, this same study showed low *Cq* variations in expression levels in different tissues and in development stages for all tested reference genes, indicating that all are suitable to be used as reference genes, even though they have not been validated. These results diverge from our data obtained by RefFinder, which shows high specificity for a particular experimental situation and how much careful analysis of candidate reference genes is required for each particular case. 

To validate the RefFinder results, the *SNF7* gene expression was investigated, showing a stable expression profile in different samples in the current study. The *SNF7* gene encodes a class E vacuolar protein conserved in several organisms, such as in the fruit fly *Drosophila melanogaster* [43], nematode *Caenorhabditis elegans* [44] and plant *Arabidopsis thaliana* [45]. This protein belongs to the ESCRT (endosomal sorting complex required for transport) complex (III) and is involved in the selection of transmembrane proteins towards to lysosomal degradation pathway [21,46] but also in multiple cell processes in Drosophila [47,48]. Given the importance of the ESCRT-III complex and its associated proteins on the regulation of membrane receptor proteins, a natural process present in several organism cells, the relative stability of *SNF7* expression in different tissues is an indication of its adequate functioning in the cellular environment. Other studies have found changing on *SNF7* expression levels *but* only after treatment with dsRNA directed to its suppression. In *Diabrotica virgifera virgifera*, the *SNF7* gene (DvSNF7) had a reduced expression profile in the carcass and midgut of neonatal larva [49] and midgut and fatty bodies of 2nd instar larva [50] only after suppression with caused dsRNA treatment directed to the *SNF7* itself, a fact not observed in cells not treated or treated with dsRNA not specific for this target gene. Similar behavior was observed in the insects *Cylas brunneus* [51] and *Cylas punctiollis* [52]. In these cases, *SNF7* expression levels remained normal in the insect body until reducing the gene expression after treatment with specific dsRNA. Such information reinforces the idea that the *SNF7* gene has an expression profile generally stable in different tissues and insects, until being suppressed by exogenous molecules.

However, for the ant *A. sexdens* we confirm that the choice of a reference gene to normalize the relative expression profile of interest genes may change the final expression outcome and should be the subject of a careful selection.

## 5. Conclusions

There is no universal reference gene suitable for all variables under analysis (development stages, larval tissues, insecticide and dsRNA treatments, etc.), since these genes can also participate in different cellular functions which implies the need for a systematic validation of possible reference genes for specific conditions. 

Finally, analyses of RT-qPCR with *A. sexdens* can be evaluated for these first-line reference genes, thus, allowing the search and study the target genes for RNAi and other biotechnology applications.

## Figures and Tables

**Table 1:** Description of the candidate reference genes and *SNF7* for RT-qPCR analysis.

Gene Abbreviation	Accession Number	Primer Sequence (Forward/Reverse 5′–3′)	Concentration (uM)	*Tm* (°C)	Amplicon (pb)	*E* (%)	*R*^2^
RPL18	EH413666	CTCTGTCGTTTCCGCTGTCTCACCTTCCGATCATGCTTATG	0.4	60.59 60.48	108	102.07	0.973
RPL32	JQ744274.1	TTCTGCCTTTCTGTTTTTCGTTTGGGTCGATAAACTGGTC	1.0	58.15 57.52	91	99.627	0.997
RPS16	EH413178.1	GAAACAAAAAGAGCCGATCCTCCACGTCCACGTTTACAAT	0.4	58.77 58.90	88	99.223	0.988
GAPDH	EH413647	CGTGGTATGACAACGAGTACGGGAGTTAGGAGGACGCAGATGAA	0.4	62.62 60.76	120	99.239	0.986
NADH	NM_001162323	GGAAAAATCGCACTAGGAGGATGTGTAGTTGCTGCTTCCATAA	0.4	60.57 58.52	137	93.529	0.99
SDHB	NM_001162436	GCTAATGTGAGCCAAAAGCCGATGCTGCGTTGTGTCATCT	0.4	59.85 59.87	139	99.346	0.984
SNF-7 ^a^	XM_012199288.1	GAGCCAACTGCTCCTTCAACTTCGACGCATTTTTCTTCG	0.4	60.31 59.12	134	91.605	0.996

^a^ Used in validation of selected reference genes.

**Table 2:** Ranking of candidate reference genes according to stability values evaluated in a pool of *A. sexdens* biological samples.

Ranking	geNorm		NormFinder		BestKeeper		Delta-*Ct*		RefFinder	
	Stability *M*-Value	Gene	Stability *SV*-Value	Gene	Stability *SD*-Value	Gene	Stability Δ*Ct*-Value	Gene	Overall Stability Value	Gene
1	0.132	GAPDH/NADH	0.056	RPS16	0.168	SDHB	0.204	RPS16	1.565	RPS16
2	-	-	0.176	NADH	0.189	RPS16	0.246	NADH	2.060	NADH
3	0.192	RPS16	0.183	SDHB	0.212	NADH	0.249	SDHB	2.213	SDHB
4	0.207	SDHB	0.188	RPL18	0.218	RPL18	0.253	RPL18	3.344	GAPDH
5	0.234	RPL18	0.207	GAPDH	0.241	GAPDH	0.260	GAPDH	4.229	RPL18
6	0.248	RPL32	0.228	RPL32	0.247	RPL32	0.275	RPL32	6.000	RPL32

Sample pool comprised forager, soldier, queen, pupa, larva, head and midgut of forager and larval midgut. Ribosomal protein L18 (*RPL18*), ribosomal protein L32 (*RPL32*), ribosomal protein S16 (*RPS16*), glyceraldehyde-3-phosphate dehydrogenase (*GAPDH*), NADH dehydrogenase (*NADH*) and succinate dehydrogenase B (*SDHB*).

**Table 3:** Ranking of candidate reference genes according to stability values evaluated in tissues of *A. sexdens.*

Ranking	geNorm		NormFinder		BestKeeper		Delta-*Ct*		RefFinder	
	Stability *M*-Value	Gene	Stability *SV*-Value	Gene	Stability *SD*-Value	Gene	Stability Δ*Ct*-Value	Gene	Overall Stability Value	Gene
1	0.079	RPL18/NADH	0.073	RPS16	0.204	RPL18	0.243	RPS16	1.414	RPL18
2	-	-	0.081	RPL18	0.208	NADH	0.244	RPL18	1.968	RPS16
3	0.121	GAPDH	0.111	GAPDH	0.226	RPL32	0.251	GAPDH	2.060	NADH
4	0.144	RPS16	0.113	NADH	0.248	GAPDH	0.270	NADH	4.000	GAPDH
5	0.176	RPL32	0.217	RPL32	0.269	RPS16	0.309	RPL32	5.045	RPL32
6	0.322	SDHB	0.599	SDHB	0.300	SDHB	0.613	SDHB	5.233	SDHB

Samples comprised three tissues such as head and midgut from the forager and larval midgut. Ribosomal protein L18 (*RPL18*), ribosomal protein L32 (*RPL32*), ribosomal protein S16 (*RPS16*), glyceraldehyde-3-phosphate dehydrogenase (*GAPDH*), NADH dehydrogenase (*NADH*) and succinate dehydrogenase B (*SDHB*).

**Table 4:** Ranking of candidate reference genes according to stability values evaluated in castes of *A. sexdens.*

Ranking	geNorm		NormFinder		BestKeeper		Delta-*Ct*		RefFinder	
	Stability *M*-Value	Gene	Stability *SV*-Value	Gene	Stability *SD*-Value	Gene	Stability Δ*Ct*-Value	Gene	Overall Stability Value	Gene
1	0.122	GAPDH/NADH	0.106	RPS16	0.182	RPS16	0.208	RPS16	1.316	RPS16
2	-	-	0.135	NADH	0.187	SDHB	0.218	NADH	1.861	NADH
3	0.189	RPS16	0.154	GAPDH	0.231	NADH	0.227	GAPDH	2.590	GAPDH
4	0.201	SDHB	0.181	RPL32	0.237	RPL32	0.245	RPL32	3.761	SDHB
5	0.222	RPL32	0.209	SDHB	0.241	GAPDH	0.257	SDHB	4.229	RPL32
6	0.237	RPL18	0.218	RPL18	0.251	RPL18	0.267	RPL18	6.000	RPL18

Samples comprised three castes such as forager, soldier and queen. Ribosomal protein L18 (*RPL18*), ribosomal protein L32 (*RPL32*), ribosomal protein S16 (*RPS16*), glyceraldehyde-3-phosphate dehydrogenase (*GAPDH*), NADH dehydrogenase (*NADH*) and succinate dehydrogenase B (*SDHB*).

**Table 5:** Ranking of candidate reference genes according to stability values evaluated in development stages of *A. sexdens.*

Ranking	geNorm		NormFinder		BestKeeper		Delta-*Ct*		RefFinder	
	Stability *M*-Value	Gene	Stability *SV*-Value	Gene	Stability *SD*-Value	Gene	Stability Δ*Ct*-Value	Gene	Overall Stability Value	Gene
1	0.163	RPL32/RPL18	0.114	RPS16	0.174	RPS16	0.261	RPS16	1.316	RPS16
2	-	-	0.208	RPL32	0.208	GAPDH	0.293	RPL32	2.115	RPL32
3	0.196	RPS16	0.213	RPL18	0.211	SDHB	0.296	RPL18	2.449	RPL18
4	0.253	SDHB	0.230	GAPDH	0.229	RPL18	0.316	GAPDH	3.557	GAPDH
5	0.285	GAPDH	0.247	SDHB	0.243	RPL32	0.329	SDHB	4.162	SDHB
6	0.308	NADH	0.293	NADH	0.274	NADH	0.353	NADH	6.000	NADH

Samples comprised three development stages such as forager, pupa and larva. Ribosomal protein L18 (*RPL18*), ribosomal protein L32 (*RPL32*), ribosomal protein S16 (*RPS16*), glyceraldehyde-3-phosphate dehydrogenase (*GAPDH*), NADH dehydrogenase (*NADH*) and succinate dehydrogenase B (*SDHB*).
